# Sociodemographic, economic, physical, and mental health status of previously hospitalised patients with COVID-19 symptoms in Bangladesh: Protocol for a mixed-method study

**DOI:** 10.3389/fpubh.2022.763812

**Published:** 2023-01-09

**Authors:** Tanvir Ahmed, Shangjucta Das Pooja, Ahmed Jojan Nandonik, Shamira Mostafa, Zarina Nahar Kabir

**Affiliations:** ^1^Research, Monitoring and Evaluation Department, SAJIDA Foundation, Dhaka, Bangladesh; ^2^Department of Neurobiology, Care Sciences and Society, Karolinska Institutet, Stockholm, Sweden

**Keywords:** long COVID, Bangladesh, COVID-19, low- and middle-income countries (LMICs), post-COVID consequences

## Abstract

**Introduction:**

Not much is known about the long-term consequences of COVID-19, popularly known as long COVID. This is particularly so in terms of patterns and clusters of symptoms over time, sociodemographic and economic characteristics of patients, and related clinical history. This is crucial for resource-constrained health systems such as Bangladesh to address long COVID as a forthcoming challenge. This protocol aims to investigate the consequences of COVID-19 over time for physical and mental health and how these are associated with demographic and socio-economic factors.

**Methods and analysis:**

This mixed-method study collected information on all patients with symptoms of COVID-19 admitted to and discharged after recovery from a COVID-19-dedicated hospital in Bangladesh (*N* = 942), from April to December 2020. The sources of data were admission records and discharge certificates from the hospital for clinical history, cross-sectional survey on physical and mental health (assessed by DASS21 scale)-related symptoms and socioeconomic changes after recovery, and qualitative in-depth interviews on experiences of COVID-19. Interviews were conducted over the phone. Quantitative analysis was done to estimate the prevalence of physical and mental health consequences of COVID-19 after recovery and the association with socio-economic and demographic information. The qualitative analysis was performed using a thematic analysis approach.

**Discussion:**

It is imperative to understand the post-COVID consequences and related health and non-health aspects to inform evidence-based policymaking, especially for resource-poor contexts such as Bangladesh. Given the dearth of evidence in this regard, the proposed study will contribute to bridging this knowledge gap. It is important to note that this study is one of the few which presents information on post-COVID-19 consequences in the context of low- and middle-income countries and the first in Bangladesh.

## Introduction

As in many low- and middle-income countries (LMICs), Bangladesh has experienced the burden of the COVID-19 pandemic. Since the identification of the first case on 8 March 2020 (1, 2), till 29 October 2022, more than two million COVID cases and more than 29,000 related deaths have been recorded in the country (3). To date, there have been a great deal of reporting and documentation on how the acute phase of the pandemic stretched the health system of Bangladesh due to the multitude of health, social, and economic challenges. However, less is known about the long-term impact of the pandemic in the country.

In 2020, a study in Bangladesh first reported that 87% of survivors of COVID-19 have at least one persistent symptom, particularly fatigue and dyspnoea (4). Later, other studies also reported that as late as 30 weeks after recovery from COVID-19, people have experienced the persistence of symptoms including considerably restricted daily activities and a range of symptoms related to respiratory distress and lethargy (5–7). In addition to the physical health-related long-term impact of COVID-19, there have been reports of considerable mental health-related symptoms. Between 2020 and 2021, several online surveys reported symptoms of various degrees (mild to severe) of depression, anxiety, and stress among people of diverse ages and social groups. These were reported to be related to a wide range of factors such as financial uncertainty, disruption in daily life including social interactions, fear of infection, disruption and inconvenience in the work environment, unemployment, being a woman, etc. (8–13). Similar evidence on considerable physical health (e.g., breathlessness, chest pain, palpitations, chronic fatigue, orthostatic dizziness, and brain fog) (14, 15) and mental health-related symptoms (stress, anxiety, fear, depression, and insomnia) (16) have been reported in studies from other countries as well. A review by the National Institute for Health Research (NIHR) suggests that about 10% of patients positive for COVID-19 continue to have at least one symptom for 12 weeks or longer. For those who stayed at home, about 20–30% experienced at least one symptom after 1 month and about 10% after 3 months. Of those who were hospitalised, between 50 and 89% had at least one symptom after 2 months. More recent evidence suggests that such symptoms of long COVID may result even after 6 months of initial recovery in people diagnosed positive for COVID-19 but had stayed home during the COVID-19 (17). Such post-COVID-19 consequences are now referred to as “long-COVID,” which essentially signifies that these post-COVID consequences are the long-term impact of COVID-19.

Considering the existing literature in Bangladesh and around the world, a few points should be noted. First, globally there is an emerging trend of long COVID, and it can be a significant challenge for the global and country-specific public health system. Second, due to the devastating impact of the pandemic, much of the focus has been on understanding and tackling the acute phase of COVID-19. As a result, little is known about the patterns and clusters of symptoms of ?long COVID” yet. Third, only a handful of studies in Bangladesh have presented evidence of mental health-related signs and symptoms of long COVID and even less on physical health-related signs and symptoms. Finally, none of the studies in Bangladesh has considered how past medical history has contributed to the consequences of COVID-19 on health. Considering this, the proposed study aimed to investigate the consequences of COVID-19 over time for physical and mental health and how these are associated with demographic and socio-economic factors. The specific objectives of the study are as follows: (1) To investigate the association between patients' socio-demographic characteristics and history of exposure to clinical symptoms of COVID-19; (2) to assess the consequences of COVID-19 on patients' physical health over time; (3) to assess the consequences of COVID-19 on patients' mental health over time; and (4) to explore the experiences of patients in terms of physical and mental health after recovering from COVID-19. It is expected that this study will contribute to developing an effective and inclusive COVID-19 mitigation plan as well as strategies and protocols for treatment and prevention of such consequences, and thereby help the health system of Bangladesh (and similar LMICs) to combat current and future similar pandemic(s).

## Methods and analysis

### Study design

This study aimed for a comprehensive understanding of the post-COVID-19 consequences among Bangladeshis based on individual post-COVID-19 physical and mental health-related experiences with relevant past clinical history and socio-economic changes. Much of this information will require an understanding of the context too. Thus, the blending of methods is deemed ideal for such complex information, popularly known as mixed-methods design. This comprises secondary data from admission records and discharge certificates from the hospital for clinical history, cross-sectional quantitative survey of physical and mental health-related symptoms and socioeconomic changes after recovery from COVID-19, and a qualitative exploration of the experience of COVID-19 and its aftermath of selected participants.

### Study period

The study was planned to be conducted between January and December 2021. However, due to ongoing COVID-19 waves in Bangladesh and intermittent lockdowns imposed by the Government of Bangladesh (GoB), there had been a considerable delay. Due to these constraints, it was expected that the data collection of the project may continue till the first quarter of 2022, including preliminary analyses of the quantitative and qualitative data.

### Study context, population, and sample

It is important to note that, in a resource-poor setting like Bangladesh, the provisions of community screening for COVID-19 and confirmatory tests like polymerase chain reaction (PCR) were scarce, especially in the 1st year of the pandemic. Also, there was a widespread stigma about COVID and reluctance toward infection prevention control and related hygiene measures in the community. As a result, the primary assumption considered for the study was “not everyone who were infected with COVID-19 has confirmation through screening and/or PCR test,” and it seemed logical to consider “hospitalised patients with COVID-19-like symptoms, admitted under physicians' advice” as the prospective sampling frame for this study. Also, this study deliberately considered symptoms of depression, anxiety, and stress, and not severe mental health conditions, as the proxy for mental health-related post-COVID-19 consequences. This is because the study was conducted amid ongoing waves of the pandemic and self-reporting information through a telephone survey, which was the only option. However, severe mental health conditions require face-to-face assessment by trained personnel based on both signs and symptoms. As mentioned in the introduction, such self-reported symptoms of depression, anxiety, and stress have been considered by others in Bangladesh and globally to document the mental health burden in relation to the COVID-19 pandemic. Therefore, symptoms of depression, anxiety, and stress as a proxy of mental health-related post-COVID-19 consequences were deemed justified for this study.

The respondents of the study were hospitalised in a COVID-19-dedicated hospital in Bangladesh with COVID-19-like symptoms. As soon as the GoB initiated the country's COVID-19 response, the SAJIDA Foundation, one of the leading non-government organizations (NGO), established a COVID-19 dedicated hospital through a Memorandum of Understanding (MoU) with the Directorate General of Health Services (DGHS), Ministry of Health and Family Welfare (MoHFW), GoB, on 21 March 2020. It was located about 16 km southeast of the capital city of Dhaka and was comprised of a four-bed intensive care unit (ICU), ventilators, dialysis units, 46-bed general ward, and 102 staff (18 doctors, 21 nurses, 5 medical technologists, biomedical engineers, support staff, management staff, and expert ICU and dialysis unit staff). The staff were trained under the direct supervision of DGHS for comprehensive and coherent hospital care for the patients of COVID-19 (18). During a period of 9 months (April to December 2020), 1,022 patients were admitted to this hospital with symptoms similar to COVID-19 infection. Unfortunately, 52 people died among them and 28 were referred to other facilities. Thus, the final sample frame of this study was 942. Box 1 shows the inclusion and exclusion criteria of the study.

Box 1 **Inclusion and exclusion criteria of the study population.**

Inclusion Criteria

- Patients with symptoms of COVID-19.
- Patients aged 18 years or older and willing to participate in the study.
- Patients who were admitted at SAJIDA COVID-19 Hospital. Exclusion Criteria
- Patients who recovered from symptoms of COVID-19 but are unable to communicate due to mental or physical challenges.

**Figure 1:**
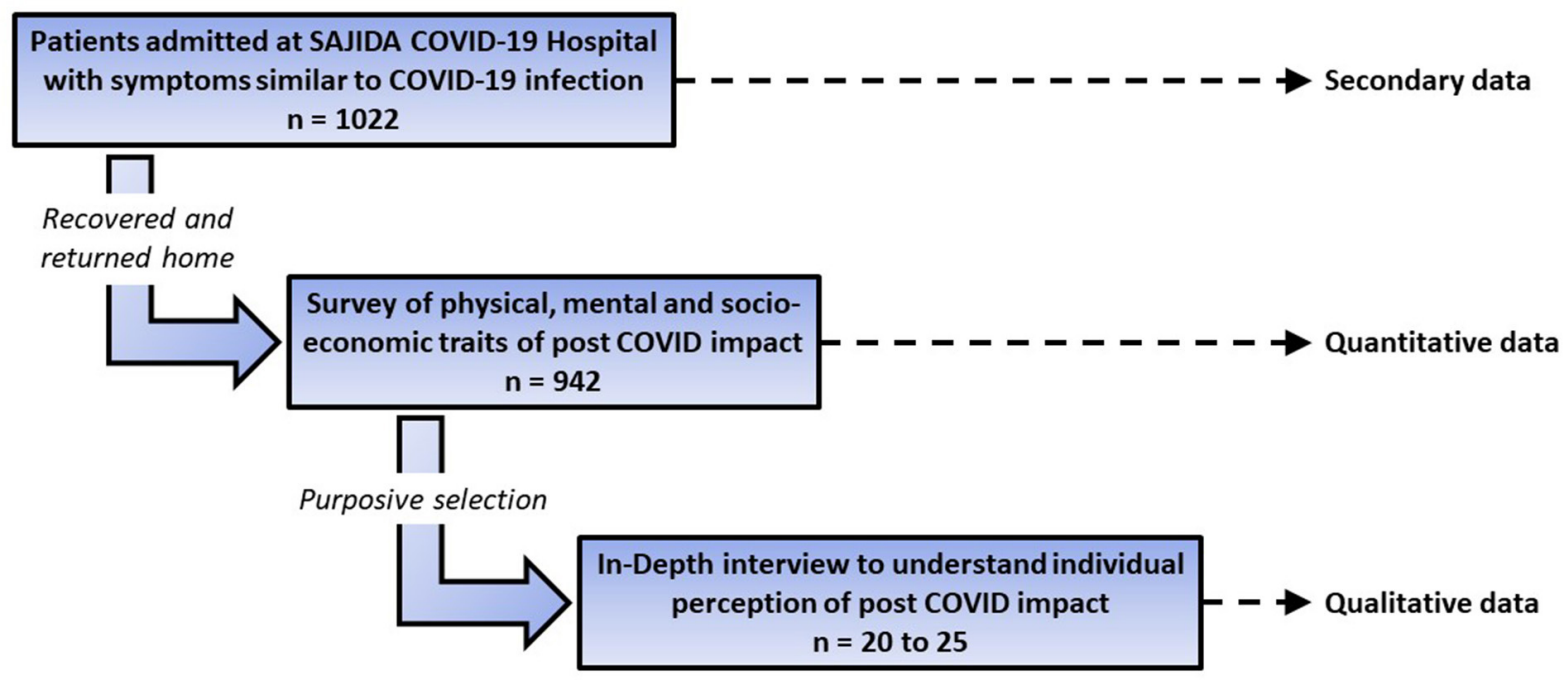
Schematic design of the proposed study.

Considering there was not enough knowledge about the prevalence of various complications (both health-related and social) after recovery from COVID-19 when the study was conceptualised, all 942 patients were considered to be the final sample with an estimated non-response rate of 50% for the quantitative survey. During the survey, at least 20 to 25 respondents who appeared to be willing and enthusiastic to tell their stories were purposively selected for a qualitative in-depth interview. However, the final number of in-depth interviewees depends on reaching data saturation. Figure 1 shows the schematic design of the study with sample and source of data.

### Data collection: Tools and techniques

The data in this study is comprised of three sources: hospital data (secondary), quantitative survey, and qualitative interviews. The hospital data were collected from the admission records and discharge certificates from April to December 2020. The quantitative survey was conducted over the telephone using a structured questionnaire. The list of telephone numbers was obtained from the hospital admission records. The list was anonymised and assigned unique identification numbers (IDs). The IDs were later used to match the demographic profile of the participants from the hospital records and to calculate the time since discharge from the hospital. The quantitative questionnaire was designed to begin with seeking informed consent. The consent was recorded and stored separately with corresponding IDs as the names of the recordings. After obtaining the consent, the rest of the questionnaire was administered. The questionnaire included three groups of questions: (a) physical health and related symptoms, (b) mental health using Depression Anxiety Stress Scales (DASS-21) (19), and (c) socioeconomic information and related changes.

The survey questionnaire was developed based on the review of literature and consultation with public health experts. The DASS-21 scale has already been translated and validated in Bangladesh (19) and deemed appropriate to assess the post-COVID-19 mental health consequences, according to a previous study. This scale includes three domains of mental health: depression, anxiety, and stress, and each domain has seven statements. The responses were recorded based on the degree of agreement with each of the statements using a Likert scale. The post-COVID-19 physical health consequences part included questions on the respiratory system and cough, the cardiovascular system such as stroke, the heart and its related diseases, the gastrointestinal system, the nervous system, and neurocognition. The data collectors were trained to record the most appropriate response to each question. The questionnaire contained a total of 148 questions and was administered after pretesting it with patients of COVID-19, who were not in the sample frame of this study. An electronic version of the final questionnaire was created using a Web-based survey tool called “Kobo Toolbox” (20). Although it is free, the privacy statement clearly states that the tool is not a commercial product and has no conflict of interest. Kobo Toolbox is funded by many humanitarian agencies including the UN. Also, it is committed to securing the privacy of the collected data and is refrained from sharing it with anyone (20). An electronic platform for data collection was chosen for two reasons: (1) to get rid of the step between data entry and data collection, thus avoiding chances of human errors during data entry and (2) to take advantage of the “skip” function in the questionnaire. Five android tablets were prepared for the data collection with seamless internet connectivity and an updated “Kobo Collect app” which contained the questionnaire (20).

The qualitative part of the study was designed to capture the experience of the respondents in relation to their post-COVID-19 physical and mental health ailments and socioeconomic challenges in greater detail and through in-depth interviews, which are deemed to be appropriate. For this, an open-ended IDI guideline was prepared. The main themes of the guidelines are shown in Box 2 and the detailed guideline has been incorporated with this manuscript as a supplementary file. This guideline was pretested through mock interviews with respondents outside the sample frame but similar to the qualitative sample of this study.

Box 2 **Guideline for qualitative interview.**

- Social and economic challenges for a person recovered from COVID-19 and coping strategy.
- Challenges of accessing healthcare for person with COVID-19.
- Actual experience and expectation about state of wellbeing during COVID-19 and after recovery.
- Use of available information in coping with challenges.

As mentioned earlier, both survey and in-depth interviews were conducted *via* telephone to remain compliant with the measures of infection prevention and related guideline. A team of seven data collectors and one supervisor were trained on the questionnaire. All are graduates and have previous experience in telephone-based data collection. The training involved interview techniques, how to obtain and record informed consent, the importance of building rapport before the interview, and using the KOBO collect app on supplied android tablets. In the practice session and pretesting, the survey interviews took about 30-40 min and qualitative interviews took about 45-60 min to complete. Since this was a telephone-based data collection (both survey and qualitative interviews), considerable non-response was expected. To keep non-response to a minimum, each respondent was reached out at least three times at various times on different days to ensure that the interviews were conducted at a time convenient to the respondents. Like the survey, in-depth interviews too were conducted with required confidentiality and only after obtaining informed consent. All interviews were audio recorded, with separate approval obtained from the respondents.

### Data management and analysis

Both quantitative and qualitative data were stored with appropriate IDs to ensure that participants remain anonymous. The codebook was accessible to the Principal Investigator and the Research Manager for future reference. The recordings of the informed consent were stored in a similar manner. During data collection, the data and consent recordings were collected in a password-protected portable hard disk by the supervisor of the data collection team and transferred to the secured server on daily basis.

Quantitative data were cleaned through frequency runs and cross-tabulation using the latest version of statistical software. Table 1 shows the types of variables and their sources. Participants' mental health status was expressed by the degree of depression, anxiety, and stress calculated by summing the scores obtained from the Likert scale response. Once cleaned, the dataset was analysed using univariate techniques to identify the distribution of the variables including measures of central tendencies. Considering that most of the variables are non-parametric, the significance of differences was assessed using *chi-square*. If and when applicable, the association between variables was analysed using bivariate and multivariate techniques such as logistic and linear regressions.

**Table 1:** Type and source of quantitative variables.

**Type of variable**	**Source**
Demographic profile (age, sex, education, location, etc.)	Secondary (hospital admission record)
Pre-COVID-19 clinical history	Secondary (hospital admission record)
Clinical history during hospitalisation	Secondary (hospital admission record and discharge certificate)
Socio-economic (household asset ownership, monthly income, asset score, occupation of the main wage earner of the household and occupation of the participant if the two are not the same, household structure, etc.)	Primary (survey)
Physical health status (respiratory and circulatory system status, gastrointestinal system-related complications, nervous system and neurocognitive complications, musculoskeletal system-related complaints, etc. during post-recovery of COVID-19)	Primary (survey)
Mental health status: the DASS-21 scale (depression, anxiety, and stress-related status during post-recovery of COVID-19)	Primary (survey)

All qualitative interviews were transcribed verbatim by trained transcribers. This includes indicating pauses and any significant noises or other verbal gestures made. The transcripts were read independently by members of the research team, and codes, categories, and themes were identified using the framework of thematic analysis (21). For this, themes in the qualitative guideline (Box 2) were followed. During the identification of the codes, weekly peer debriefing meetings were conducted to exchange ideas. This helps to identify any emerging codes/categories/themes. Once completed, the categories and themes were organised sequentially to discover typical and atypical patterns, which were later reported with appropriate contextualization.

### Limitations of the study

The main limitation of the study is that it was based on self-reported data, which may result in some recall bias considering the time gap between discharge from the hospital and data collection. To minimise this, clear articulation of the questions, considerable probing, repeated attempts, and referencing to relevant incident/historical milestones were made and the questionnaire was administered by trained and experienced data collectors. This helps in ensuring that the participants have a clear understanding of the questions and thus their responses have higher validity and reliability.

## Discussion

There is increasing evidence that the burden of the COVID-19 pandemic extends beyond its acute phase and that the LMICs are more vulnerable considering their resource-constrained health system and existing public health challenges compared to the high-income countries (HICs) (1, 22). A comprehensive response to the COVID-19 pandemic requires both short-term and long-term strategies. In the short term, considerable evidence exists on the effectiveness of individual- and community-level behavioural measures in managing the acute phase, vaccine technologies, and restoration of the usual health and other development activities. For long-term strategies, it is imperative to understand how post-COVID-19 consequences can be managed by countries with limited resources, like Bangladesh. As highlighted in the introduction, the primary basis of any health system response should be a clear understanding of what the post-COVID-19 consequences are, who are affected by those, and what can be done to help the people and groups who are being affected.

In the current context, there is a considerable pool of literature to establish the emergence of post-COVID-19 consequences, which is now called long COVID. However, there is a pronounced gap between HICs and LMICs, considering the plan to deal with long COVID as an impending public health challenge. A good example can be the United Kingdom, where public health researchers have been able to identify (to some extent) how long COVID is affecting the population and how it can be managed at various levels of the healthcare system, including primary healthcare (23, 24). In contrast, countries like Bangladesh lack many aspects of knowledge regarding post-COVID-19 consequences. To date, only a handful of studies have been able to show the post-COVID-19 mental health-related consequences in Bangladesh. The evidence is even slimmer when it comes to post-COVID-19 physical health-related consequences. The literature review for the conceptualisation of this study could identify only three of such studies. With such limited knowledge, it is unlikely to recognise the extent of the challenges of post-COVID-19 consequences in Bangladesh and devise appropriate provision of service at various levels of healthcare.

Given the need and dearth of evidence, the proposed research can contribute to bridging this knowledge gap. The project stands on the strength of both clinical and self-reported data at the household level. Therefore, it will be able to look at post-COVID-19 consequences from both socioeconomic as well as medical (physical and mental) dimensions. Furthermore, the mixed-method design of the study is also an opportunity to consider post-COVID-19 consequences from both subjective and objective viewpoints. With the available fund, the project will consider future rounds of observations; therefore, it has the potential to emerge as a cohort with scope for future health and social interventions to design and deliver required healthcare for “long COVID” in Bangladesh and by extension to other similar LMICs.

## Ethics statement

This study involves human participants and has been reviewed and approved by Bangladesh Medical Research Council (BMRC) Reference No. 35115102020. All patients/participants provided their written informed consent to participate in this study.

## Author contributions

The study was conceived, planned, and designed by ZNK, SDP, and AJN. TA, SDP, AJN, and ZNK participated in the training of the data collectors of the study and the revision of the data collection instruments. TA developed the manuscript with contributions from all the co-authors. Ethical approval for the study was processed by all the authors. All authors read and approved this protocol for publication.
